# Evaluation of Gram Negative Bacterial Contamination in Dental Unit Water Supplies in a University Clinic in Tabriz, Iran

**DOI:** 10.5681/joddd.2011.021

**Published:** 2011-09-05

**Authors:** Firoz Pouralibaba, Esrafil Balaei, Atabak Kashefimehr

**Affiliations:** ^1^Assistant Professor, Department of Oral Medicine, Faculty of Dentistry, Tabriz University of Medical Sciences, Tabriz, Iran; ^2^DDS, Department of Community Dentistry, Faculty of Dentistry, Tabriz University of Medical Sciences, Tabriz, Iran; ^3^Assistant Professor, Department of Periodontics, Faculty of Dentistry, Tabriz University of Medical Sciences, Tabriz, Iran

**Keywords:** CFU, Dental unit, Gram-negative bacteria, water sources

## Abstract

**Background and aims:**

Bacterial contamination of dental unit water supplies (DUWS) has attracted a lot of attention in recent years due to the emergence of serious infectionsin susceptible dental patients. The aim of the present study was to evaluate the presence of gram-negative bacterial contamination in DUWS at Tabriz University of Medical Sciences Faculty of Dentistry.

**Materials and methods:**

This descriptive study was carried out on 51 active dental units in different departments. Con-tamination was determined by taking samples from the unit's water supply before dental procedures and the use of specific culture media. The cultures were evaluated after 48 hours.

**Results:**

Gram-negative bacterial contamination was identical in all the departments. In the departments on the ground floor, namely Departments of Periodontics and Oral and Maxillofacial Surgery, Pseudomonas contamination was observed in 71% of units; in the departments on the first floor, namely Departments of Prosthodontics, Orthodontics and Pedodon-tics, 46.8% of the units had Pseudomonas contamination; and in the departments on the second floor, namely Departments of Operative Dentistry and Endodontics, 37.7% of the units demonstrated Pseudomonas contamination.

**Conclusion:**

Gram-negative bacterial contamination was evident in the evaluated DUWS. The contamination type was identical but the number of contaminated units decreased with the increase in the height of the floors.

## Introduction

Infection control which is of utmost importance, came to attention in dentistry in the control of hepatitis virus and HIV infections. Research on the topic of infection and its transmission has a great role in the introduction of measures to control it.^1^ Subsequent to a study carried out by Murray & Slack on dental water/air syringe in 1957 and a report on its contamination, Ino Sciaky reported staphylococcal contamination of dental unit water supplies (DUWS) in 1962.^2^

Contamination of DUWS is a well-known subject.^1^ Some microorganisms implicated in the contamination of DUWS include gram-positive bacteria such as Streptococcus hemolyticus and Staphylococcus aureus, and gram-negative bacteria such as Pseudomonas, Legionella, and coliforms bacteria.^3 - 13^ Some studies have reported bacterial contamination up to 9000000 CFUs.^4^

Studies have focused on the role of biofilms in the spread of infection.^1^ Biofilms are microbial populations which adhere to surfaces and are mostly located at the liquid-surface interface. They predominantly consist of bacteria and other microorganisms in a matrix of polymers derived from the environment and the microorganism itself. Biofilm microorganisms have a greater chance of survival compared to microorganisms in water and planktons. They are also more resistant to antibiotics and agents capable of destroying planktons. Therefore, in the control of biofilms a general approach should be considered.

Aqueous environments surfaces are exposed to a large number of bacteria. The presence of biofilms in urban water pipes and in dairy products factories have been reported. ^3 ,14 ,15^ Dental plaque is a biofilm consisting of oral bacteria in a matrix of bacterial extracellular polysaccharides and salivary glycoproteins.^16^ Metabolism of plaque bacteria results in dental caries. Bacterial endocarditis is a result of bacterial growth in the biofilm adhering to the endothelium of heart values; these bacteria might have originated from the oral cavity.^17^

Biofilms are also found on a large number of devices and instruments including intravenous catheters, injection needles, urinary catheters, intrauterine devices (IUDs), cardiac pacemakers and articular prostheses.^18^ Bacterial biofilms on medical instruments and devices are rather resistant to antibiotics and are therefore a source for recurrent infections. American dental association (ADA) had recommended that until the year 2000, bacterial contamination of DUWS should not exceed 200 CFUs/mL. ADA guidelines included the provision of a separate water reservoir other than the urban water source (involving alterations in the design of dental units), the use of disinfecting agents in the tubes, daily evacuation of water tanks, the use of filters, flushing of the tubes for a few minutes before dental procedures, autoclaving of handpieces, and the use of UV light to control. ^19- 33^

In any setting, measures should be adopted to prevent infections in susceptible individuals subsequent to dental treatments,^34^ and identification of potent microorganisms as a source for potentially dangerous infections should be one of the major aims of infection control programs in a society.^1 , 4^ Epidemiologic studies in different countries have yielded conflicting results on the subject. Most of the microorganisms found in DUWL are, however, Gram-nagative, heterotrophic bacteria.^35^ The aim of the present study was to evaluate the presence of gram-negative bacterial contamination in the DUWS, i.e. water/air syringe and handpiece outlets, at a university clinic in Tabriz, Iran.

## Materials and Methods

All dental units at Tabriz University of Medical Sciences Faculty of Dentistry were examined. Only active units with working water/air syringes and handpiece outlets were included in the study.

Samples were taken on the first day of the week before the start of the working hour. After 2 minutes flushing, 5 mL of water from the water/air syringe and the handpiece outlet was taken using 5-mL sterile test tubes containing nutrient broth. Sterile water was used as negative control to evaluate lack of cross contamination during sampling.

All the samples were sent to the Department of Microbiology at the Faculty of Medicine for microbiologic evaluation. Samples were then incubated for 24 hours; subsequently, the samples were transferred to specific culture media namely EMB (gelose containing eosine and methylene blue) and McCankey (containing lactose with colored pH reagent) and incubated for 48 hours at 37˚C. The samples were evaluated twice, 24 and 48 hours after incubation. The samples in EMB culture media formed large blue-colored colonies; the samples in McCankey culture media formed small separated colonies. Differential culture media were then used to identify the colonies, which included Simian citrate, Glickler iron agar, SIM (SH_2_, Indole, Motility), MR, VP and urease. In addition, oxidase, catalase and motiliy tests were carried out. Pseudomonas colonies were identified by positive oxidase and catalase tests and positive motility test.

## Results

**Figure 1:**
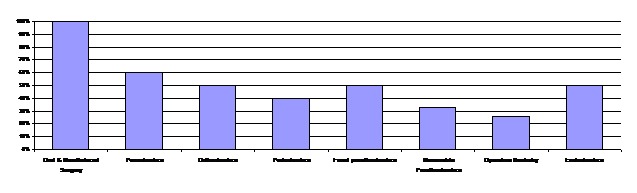
Comparison of contaminated dental unit water supplies in the departments evaluated.

**Figure 2:**
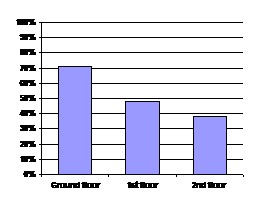
Distribution of contaminated dental unit water supplies on different floors.

The contamination levels of DUWS in the departments evaluated and according to the floor are presented in Figures 1 and 2.

In the departments located on the ground floor, including the Departments of Periodontics and Oral and Maxillofacial Surgery, 71% of 14 active units were contaminated. In the departments of the first floor, including the Departments of Prosthodontics, Orthodontics and Pedodontics, 46.8% of 23 active units were contaminated. Of 14 dental units on the second floor in Departments of Operative Dentistry and Endodontics: 35.7% of 14 active units were contaminated.

Evaluating different parts of dental units, 47% of water/air syringes and 43% of handpiece outlets were found to be contaminated.

## Discussion

Suction and back-flow of patients’ saliva occurs through saliva ejector or handpiece outlet into the water pipes of the dental unit; furthermore, stable microbial environments deposited in the unit water pipes as biofilms act as potential foci for infection. In the present study, of the 51 dental units evaluated, 26 units were contaminated with pathogenic microorganisms. The results of the present study showed that contamination of water/air syrintes with gram-negative bacteria was more than that of handpiece outlets, which might be attributed to the greater role of biofilms. It is expected that higher floors should have higher contamination rates because of greater stasis of water on those floors; however, the results showed the opposite. It seems that flushing of the outlets in the Departments of Operative Dentistry and Endodentics on the third floor had a great role in the lower rate of contamination in these departments, while lack of daily use of water/air syringes and handpieces in the Departments of Periodontics and Oral and Maxillofacial Surgery has played a role in the establishment of contamination.

The results of the present study are consistent with the results of a previous study, reporting the presence of Pseudomonas in two evaluations.^10^ However, the latter study did not use sampling tubes and specific culture media. Similar results were reported using swabs for sampling and providing smears for non-specific culture media,^4^ which is different from the method used in the present study.

Other studies using specific culture media have also reported similar results, but have not determined the rate of gram-negative bacterial contamination separately for each department. Following the death of a dental practitioner as a result of dental unit contamination with Legionella in 1995, the ADA guidelines for controlling dental unit contamination was issued in the same year, which included some instructions for dental unit manufacturers. The role of designing the complex structure of dental units in the rate of contamination has been established,^36^ but this was not taken into account in the present study. The presence of biofilms in the dental unit water pipelines is an established fact;^35^ therefore, the guidelines issued by ADA to reduce infection risk in the elderly, organ transplant patients, patients receiving immunosuppressive medications, patients with asthma or chronic pulmonary conditions and patients with AIDS should be observed.

In addition, the dental team rendering treatment, which consists of dental practitioners and dental assistants, are at a risk for infections. It has been shown that Legionella antibody level in dental practitioners is significantly higher than that in the general population.^37^

The following considerations are recommended to reduce the risk of infection transmission:

1. Application of the issued guidelines, including placement of filters and the use of disinfecting agents in the dental unit water pipelines in a periodic manner.
2. Modifications in dental unit designing and the use of a separate water tank with the capacity for daily evacuation.
3. Flushing of the outlets before and after dental procedures for 2–3 minutes to prevent back-flow.
4. Observation of principles of sterilization for handpieces, turbines and ultrasonic devices.
5. Use of sterile water or physiologic serum during surgeries involving bone.
